# Association of triglyceride glucose index with cardiovascular events: insights from the Isfahan Cohort Study (ICS)

**DOI:** 10.1186/s40001-024-01728-4

**Published:** 2024-02-17

**Authors:** Hamed Rafiee, Noushin Mohammadifard, Fatemeh Nouri, Ghazaal Alavi Tabatabaei, Jamshid Najafian, Masoumeh Sadeghi, Maryam Boshtam, Hamidreza Roohafza, Fahimeh Haghighatdoost, Razieh Hassannejad, Nizal Sarrafzadegan

**Affiliations:** 1 Isfahan Cardiovascular Research Center, Cardiovascular Research Institute, Isfahan University of Medical Sciences, Isfahan, Iran; 2 Pediatric Cardiovascular Research Center, Cardiovascular Research Institute, Isfahan University of Medical Sciences, Isfahan, Iran; 3 Hypertension Research Center, Cardiovascular Research Institute, Isfahan University of Medical Sciences, Isfahan, Iran; 4 Cardiac Rehabilitation Research Center, Cardiovascular Research Institute, Isfahan University of Medical Sciences, Isfahan, Iran; 5 Heart Failure Research Center, Cardiovascular Research Institute, Isfahan University of Medical Sciences, Isfahan, Iran; 6 Interventional Cardiology Research Center, Cardiovascular Research Institute, Isfahan University of Medical Sciences, P. O. Box, Isfahan, 81745-15 Iran; 7 Faculty of Medicine, School of Population and Public Health, University of British Columbia, Vancouver, Canada

**Keywords:** Cardiovascular disease, Coronary heart disease, Triglyceride glucose index

## Abstract

**Background:**

There is limited evidence regarding the evaluation of the association between the triglyceride glucose (TyG) index, an indicator of insulin resistance, and the incident risk of cardiovascular disease (CVD). Therefore, we aimed to examine the relationship between the TyG index and CVD incidence in a cohort of Iranian adults.

**Methods:**

This study was performed in the framework of the Isfahan Cohort Study (ICS). The study population included 5,432 individuals aged ≥ 35 years. CVD events, including acute myocardial infarction (MI), stroke, and unstable angina (UA), were diagnosed by physicians. The TyG index was calculated as Ln [fasting triglycerides (mg/dL) × fasting plasma glucose (mg/dL)/2]. The relationship between the TyG index and CVD events was investigated using Cox regression models. Receiver operating characteristics (ROC) curve analysis was used to determine the best cut-off for the TyG index for predicting CVD outcomes.

**Results:**

During a median follow-up period of 11.2 years, a total number of 819 CVD, 164 MI, 172 stroke, and 384 UA were recorded. Following adjustment for multiple confounders, elevated TyG levels were associated with a higher risk of CVD (HR = 1.48; 95% CI 1.22–1.79; *p* < 0.001), MI (HR = 2.24; 95% CI 1.42–3.52; *p* < 0.001), stroke (HR = 1.45; 95% CI 0.96–2.19; *p* = 0.042), but not UA (HR = 1.28; 95% CI 0.96–1.69). The optimal TyG index cut-off was 8.91 for predicting CVD (sensitivity 58%; specificity 58%), 9.04 for predicting MI (sensitivity 57%; specificity 65%), 8.92 for predicting stroke (sensitivity 57%; specificity 57%), and 8.98 for predicting UA (sensitivity 53%; specificity 61%).

**Conclusion:**

We found a robust, direct association between the TyG index and the incidence of CVD events. This emphasizes the significance of observing the TyG index as an indicator of the occurrence of CVD events.

## Background

Cardiovascular diseases (CVDs) have emerged as a significant global health concern, resulting in a substantial number of deaths in recent years [1]. With an estimated annual mortality of 17.9 million individuals, CVDs rank as the leading cause of death worldwide [2, 3]. Likewise, CVDs constitute a leading cause of both mortality and morbidity in the Middle East and North Africa region [4], necessitating the identification and management of CVD risk factors.

Insulin resistance (IR) is a well-established risk factor and a recognized pathophysiological pathway in the development of CVDs [5]. Although the reference method for measuring IR is the hyperinsulinemic-euglycemic clamp (HIEC), its applicability in population-based research is hindered by its complexity and cost [6]. The homeostasis model assessment of IR (HOMA-IR) serves as the most commonly used surrogate method for assessing IR. However, it exhibits limitations in individuals receiving insulin treatment or those with compromised beta cell function [7]. Another simple and reliable surrogate method is the triglyceride glucose (TyG) index [8, 9]. The TyG index is derived from logarithmic calculations involving triglyceride (TG) and fasting plasma glucose (FPG) levels, and its validity has been evaluated against reference measurement methods of IR in various studies [10]. A systematic review reported that the sensitivity and specificity of the TyG index range from 67 to 96% and from 32.5% to 99.7%, respectively [10].

IR is a prevalent metabolic disorder within the Iranian population [11] and represents a robust predictor of CVD events [5]. Although the association between the TyG index, as a surrogate marker of IR, and the incidence of CVD events has been examined in multiple cohort studies [12–16], only two studies within Iranian cohorts have explored this relationship to date [17, 18]. However, these studies come with some limitations that restrict the external validity of their results. For instance, the populations of these studies were restricted to one [17] or three [18] specific area(s) of the metropolitan cities of Tehran and Mashhad or followed participants for a short duration with a limited number of events [18]. To bridge this knowledge gap, our research endeavors to explore the correlation between the TyG index and cardiovascular disease (CVD) events through a prospective cohort study carried out in both urban and rural regions of three central Iranian cities.

## Methods

### Study population

The Isfahan Cohort Study (ICS) data were used in this study's secondary analysis. The ICS is a population-based prospective cohort study that commenced in 2001 and currently includes 6504 individuals, comprising 3168 men and 3336 women, aged 35 years or older [19]. Using a stratified cluster random sampling technique, the participants were chosen from three regions in central Iran: 3323 from Arak, 2153 from Isfahan, and 1028 from Najaf-Abad. Inclusion criteria included age ≥ 35, Iranian nationality, and mental competence. Exclusion criteria included pregnancy and a history of MI, stroke, or heart failure. Detailed information on the study methodology has been previously provided [19]. During the baseline visit, demographic characteristics and socioeconomic status were collected through face-to-face interviews. Additionally, physical examinations, anthropometric measurements, and blood sampling were performed following standardized methods.

At baseline, all subjects were free from any CVD events. Over the follow-up period, the incidence of CVDs was monitored through phone calls every two years, and the occurrence of CVD events was recorded when reported. Furthermore, all measurements conducted at baseline were repeated every 6 years, specifically in 2007 and 2013.

For this study, we utilized data from 5432 participants who underwent measurements both in 2007 and 2013 and who provided complete data on the variables of interest, namely, TG, FPG, and CVD events. Before their participation, all individuals provided written informed consent, and the study obtained ethical approval from the Ethics Committee of the Research Council at the Isfahan Cardiovascular Research Center, a partnering institution with the World Health Organization located in Isfahan, Iran. The study protocol adhered to the principles outlined in the Helsinki Declaration.

### Data collection

- Demographic variables
  During the enrollment procedure, 30-min home interviews were conducted by trained health experts using a comprehensive questionnaire. This questionnaire gathered information on demographic and socioeconomic variables, including age, sex, marital status, level of education, and residency location. Lifestyle characteristics such as smoking status and dietary intake, as well as medical history related to hypertension, diabetes mellitus, and dyslipidemia, were also recorded [20, 21]. The physical activity level of the participants was evaluated using the Persian version of the International Physical Activity Questionnaire (IPAQ), which has been validated [22]. Dietary intakes were evaluated using a validated qualitative, 48-item food frequency questionnaire (FFQ) [23].
- Clinical and anthropometric measurements
  Following the interviews, participants underwent a 20-min physical examination conducted by trained staff. During this examination, blood pressure measurements and anthropometric variables, including height, weight, and waist circumference, were obtained. These measurements were performed following standardized protocols and using calibrated instruments [24, 25].
- Laboratory measurements
  Blood samples (10 mL) were drawn from each participant after a 12-h fast. Laboratory measurements of the samples were conducted at the central lab of the Isfahan Cardiovascular Research Center, following external national and international quality requirements. An autoanalyzer (Eppendorf, Hamburg, Germany) was used to quantify FPG, TG, and serum total cholesterol enzymatically [26]. Following the precipitation of very low-density and low-density lipoproteins with dextran sulfate magnesium, blood high-density lipoprotein cholesterol (HDL-C) was tested [27]. Serum low-density lipoprotein cholesterol (LDL-C) was measured using the Friedewald equation for cases with TG < 400 mg/dL and standard kits for cases with TG > 400 mg/dL.

### Definition of terms

The TyG index was calculated as Ln (fasting TG [mg/dL] × FPG [mg/dL]/2). The body mass index (BMI) was determined by dividing the weight (kg) by the square of height (m^2^). The global dietary index (GDI) was calculated as a measure of food quality. For this purpose, 29 food items were categorized into seven groups, and each group was assigned a score of 0, 1, or 2 based on its frequency in the participant’s dietary intake. For the healthy food groups, the group score increased with its consumption by the subject, but unhealthy groups received lower scores the more they were used. The scores of the seven food categories were summed to calculate the final GDI, with a smaller value indicating a healthier diet.

Hypertension was defined as meeting one of the following criteria: having a systolic blood pressure (SBP) ≥ 140 mmHg, a diastolic blood pressure (DBP) ≥ 90 mmHg, or being on antihypertensive medication. Diabetes mellitus was diagnosed in patients who exhibited FPG ≥ 126 mg/dL, 2-h postprandial glucose (2hPG) ≥ 200 mg/dL, or were currently taking anti-diabetic medications.

Acute myocardial infarction (MI) was diagnosed if two or more of the following criteria were present: (1) typical chest pain lasting more than 30 min; (2) ST elevation > 0.1 mV in at least two adjacent electrocardiograph leads; and (3) increased levels of cardiac markers in the patient's serum, including creatine kinase (CK), creatine kinase-myoglobin binding (CK-MB), CK-MB mass (CK-MBm), or troponin (cTn). Stroke was characterized as a focal neurological disorder of abrupt onset that persisted for a minimum of 24 h, with a likely vascular origin, following the definition established by the World Health Organization [28]. Unstable angina (UA) was defined as an episode of chest pain exceeding 20 min within 24 h before hospital admission. This episode needed to deviate from the usual pattern of the patient’s angina or pain, manifesting an elevating pattern and a high intensity, and being described as a frank pain [29]. CVDs were defined as a combination of stroke and ischemic heart disease.

### Follow-up and case ascertainment

Participants were followed up through phone calls every 2 years, and home visits were conducted for nonresponders after four unsuccessful phone call attempts. During each phone call, participants were asked to report their current vital status, any history of hospitalization (especially for cardiovascular causes), and any symptoms related to stroke. These symptoms encompassed hemiparesis, dysarthria, facial asymmetry, imbalance, or temporary monocular blindness. In the event of any of these occurrences, the date of the event, physician diagnosis, hospital name, and a relevant questionnaire were documented and recorded.

All measurements obtained at baseline were repeated in 2007 and 2013. During these interviews, participants were asked about any history of hospitalizations (with a focus on coronary and cerebrovascular events), the physician’s diagnosis, and the hospital name. The follow-up was concluded for subjects with CVD, and they were tracked only for mortality. The accuracy of reported data regarding CVD events was verified using the registry database of the Surveillance Department at the Isfahan Cardiovascular Research Center.

### Statistical analysis

Baseline characteristics of participants were compared between males and females using an independent sample *t* test (or Mann‒Whitney test, as needed) for continuous variables and a Chi-square test for categorical variables. To assess the differences in baseline characteristics among the tertiles of the TyG index, we conducted a comparative analysis using analysis of variance (ANOVA) for continuous variables and the Chi-square test for categorical variables. In cases where the assumptions were not met, we utilized the Kruskal‒Wallis test. Continuous variables are presented as the mean ± SD, and categorical variables are described as numbers (percentages).

We calculated person-years of follow-up starting from the recruitment date until the occurrence of the first recorded event. Crude and multiple-adjusted hazard ratios (HRs) and 95% confidence intervals (CIs) for the association between the TyG index and cardiovascular events were calculated using Cox proportional hazards regression. Model 1 was unadjusted, and model 2 was controlled for age at baseline and sex. Model 3 was additionally adjusted for education, marital status, and residency area. Model 4 was additionally controlled for GDI, smoking status (never smoked/ever smoked), and total daily physical activity (MET-min/day). The final model was further adjusted for risk factors, including BMI, hypertension, and elevated total cholesterol.

We used the receiver operating characteristics (ROC) curve analysis to evaluate the discriminative capacity of the TyG index to predict the occurrence of various outcomes. The area under the curve (AUC) was used as a global measure of diagnostic accuracy. The interpretation of AUC scaled as follows: under 0.5 indicated a futile test, between 0.5 and 0.6 demonstrated bad diagnostic precision, the 0.6–0.7 range suggested sufficient accuracy, 0.7–0.8 denoted good accuracy, the 0.8–0.9 range signified very good accuracy, and excellent acuity was represented by 0.9–1.0 [30]. The best cut points for the TyG index were determined based on the maximum value of (sensitivity × specificity) according to the method proposed by Liu [31].

All analyses were done using SPSS version 20 and STATA version 14.0, considering a significance level of *p* < 0.05.

### Use of generative AI and AI-assisted technologies in the writing process

The authors utilized the linguistic revision capabilities of chatGPT-3.5 during the manuscript preparation process. Subsequently, they carefully examined and modified the content as needed, assuming complete responsibility for the publication's content.

## Results

Table 1 illustrates the baseline characteristics of the study participants. A total of 5432 individuals were enrolled in this study, comprising 2648 (48.75%) males and 2784 (51.25%) females. The average age of the participants was 50.69 ± 11.62 years with a marginally higher mean age observed in males (51.15 ± 11.92 years) compared to females (50.26 ± 11.32 years) (*p* = 0.025). It was observed that men had a higher prevalence of current smoking than women (*p* < 0.001). Women, on the other hand, exhibited higher rates of hypertension, diabetes mellitus, dyslipidemia, and excess body weight (*p*-values ranged from < 0.001 to 0.012). Furthermore, women had significantly elevated levels of total cholesterol, LDL cholesterol, and HDL cholesterol compared to men (*p* < 0.001).

**Table 1:** Participants’ baseline characteristics

		Overall (*n* = 5432)	Male (*n* = 2648)	Female (*n* = 2784)	*p* value^†^
Triglyceride glucose index		8.87 (0.60)	8.88 (0.59)	8.87 (0.60)	0.266
Age, years		50.69 (11.62)	51.15 (11.92)	50.26 (11.32)	0.025
Education	0–5 years	3861 (71.2)	1648 (62.4)	2213 (79.6)	< 0.001
	6–12 years	1230 (22.7)	729 (27.6)	501 (18.0)	
	> 12 years	333 (6.1)	266 (10.1)	67 (2.4)	
Marital status, married		4951 (91.1)	2605 (98.4)	2346 (84.3)	< 0.001
Smoking status	Current	846 (15.6)	792 (29.9)	54 (1.9)	< 0.001
	Former	353 (6.5)	312 (11.8)	41 (1.5)	
	never	4233 (77.9)	1544 (58.3)	2689 (96.6)	
Hypertension		1510 (27.8)	653 (24.7)	857 (30.8)	< 0.001
Diabetes		459 (8.4)	198 (7.5)	261 (9.4)	0.012
Dyslipidemia		4735 (87.2)	2178 (82.3)	2557 (91.8)	< 0.001
BMI, kg/m^2^		26.77 (4.68)	25.54 (3.92)	27.95 (5.03)	< 0.001
Waist-to-hip ratio		0.93 (0.08)	0.93 (0.08)	0.93 (0.09)	0.002
Systolic blood pressure, mm Hg		121.64 (20.95)	121.09 (20.14)	122.15 (21.67)	0.368
Diastolic blood pressure, mmHg		78.40 (11.52)	78.08 (10.86)	78.70 (12.10)	0.486
Fasting glucose, mg/dL		88.67 (32.84)	88.11 (32.77)	89.20 (32.91)	0.303
Total cholesterol, mg/dL		214.12 (52.25)	208.13 (51.54)	219.81 (52.30)	< 0.001
LDL cholesterol, mg/dL		128.96 (43.42)	123.83 (43.03)	133.84 (43.22)	< 0.001
HDL cholesterol, mg/dL		46.91 (10.36)	45.36 (10.06)	48.39 (10.43)	< 0.001
Triglycerides, mg/dL		191.28 (103.26)	194.68 (106.88)	188.04 (99.61)	0.074

Values are *n* (%) for categorical variables and mean (SD) for continuous variables

*BMI* body mass index, *LDL* low-density lipoprotein, *HDL* high-density lipoprotein

^†^Derived from either an independent sample *t* test or a Mann–Whitney test for continuous variables and a Chi-squared test for categorical variables

Table 2 displays the hazard ratios for associations between tertiles of the TyG index and risk of cardiovascular outcomes, including CVD, MI, stroke, and UA. The analysis records 819 incidents of CVD, 164 of MI, 172 of stroke, and 384 of UA. Across progressively higher TyG tertiles, there were significantly increased risks for developing all cardiovascular endpoints. For CVD, the highest TyG tertile showed a 48% higher hazard compared to the lowest tertile (HR 1.48; 95% CI 1.22–1.79. There was also a significant dose–response trend of higher CVD risk with increasing TyG levels (*p* < 0.001). The middle TyG tertile also had elevated CVD risk, indicating the CVD relationship tracks closely in a linear fashion with TyG. Similarly for MI, the top TyG tertile had over twofold higher hazard versus the bottom tertile (HR 2.24; 95% CI 1.42–3.52). The trend was likewise significant (*p* < 0.001), confirming a graded response. Stroke hazard was also 45% higher in the highest versus lowest TyG tertile, demonstrating a significant trend (*p* = 0.042). UA showed a borderline risk increase of 28% in the top tertile, while the trend was just marginally significant in the fully adjusted model.

**Table 2:** Association of triglyceride glucose index with clinical outcomes by tertiles

	Model 1	Model 2	Model 3	Model 4	Model 5
Cardiovascular disease					
Tertile 1	1 (Ref.)	1 (Ref.)	1 (Ref.)	1 (Ref.)	1 (Ref.)
Tertile 2	1.34 (1.11–1.61)	1.25 (1.04–1.51)	1.25 (1.04–1.51)	1.27 (1.05–1.53)	1.14 (0.93–1.38)
Tertile 3	1.91 (1.60–2.27)	1.73 (1.46–2.07)	1.71 (1.44–2.04)	1.70 (1.42–2.02)	1.48 (1.22–1.79)
* P* for trend^†^	< 0.001	< 0.001	< 0.001	< 0.001	< 0.001
Myocardial infarction					
Tertile 1	1 (Ref.)	1 (Ref.)	1 (Ref.)	1 (Ref.)	1 (Ref.)
Tertile 2	1.62 (1.03–2.54)	1.55 (0.98–2.43)	1.53 (0.97–2.40)	1.58 (1.01–2.48)	1.45 (0.91–2.32)
Tertile 3	2.64 (1.75–3.99)	2.46 (1.63–3.72)	2.45 (1.62–3.71)	2.52 (1.66–3.82)	2.24 (1.42–3.52)
*P* for trend^†^	< 0.001	< 0.001	< 0.001	< 0.001	< 0.001
Stroke					
Tertile 1	1 (Ref.)	1 (Ref.)	1 (Ref.)	1 (Ref.)	1 (Ref.)
Tertile 2	1.14 (0.75–1.72)	1.04 (0.69–1.58)	1.04 (0.69–1.57)	1.06 (0.70–1.61)	0.99 (0.64–1.53)
Tertile 3	1.83 (1.26–2.64)	1.63 (1.12–2.36)	1.55 (1.07–2.25)	1.55 (1.07–2.26)	1.45 (0.96–2.19)
P for trend^†^	0.001	0.005	0.011	0.012	0.042
Unstable angina					
Tertile 1	1 (Ref.)	1 (Ref.)	1 (Ref.)	1 (Ref.)	1 (Ref.)
Tertile 2	1.35 (1.03–1.77)	1.27 (0.69–1.67)	1.27 (0.97–1.67)	1.25 (0.95–1.64)	1.07 (0.81–1.43)
Tertile 3	1.79 (1.39–2.31)	1.62 (1.26–2.09)	1.61 (1.25–2.08)	1.55 (1.19–2.00)	1.28 (0.96–1.69)
P for trend^†^	< 0.001	< 0.001	< 0.001	0.001	0.062

Model 1: crude

Model 2: adjusted for age (years) and sex (man/woman)

Model 3: additionally, adjusted for education level (0–5 years/6–12 years/ > 12 years), marital status (married/not married), and residency location (urban/rural)

Model 4: additionally adjusted for global dietary index (GDI), smoking status (never smoked/ever smoked), and total daily physical activity (MET-min/day)

Model 5: additionally, adjusted for BMI (kg/m^2^), hypertension (yes/no), and high total cholesterol (yes/no)

^†^Derived from a Mantel‒Haenszel extension for Chi-square test

**Fig. 1:**
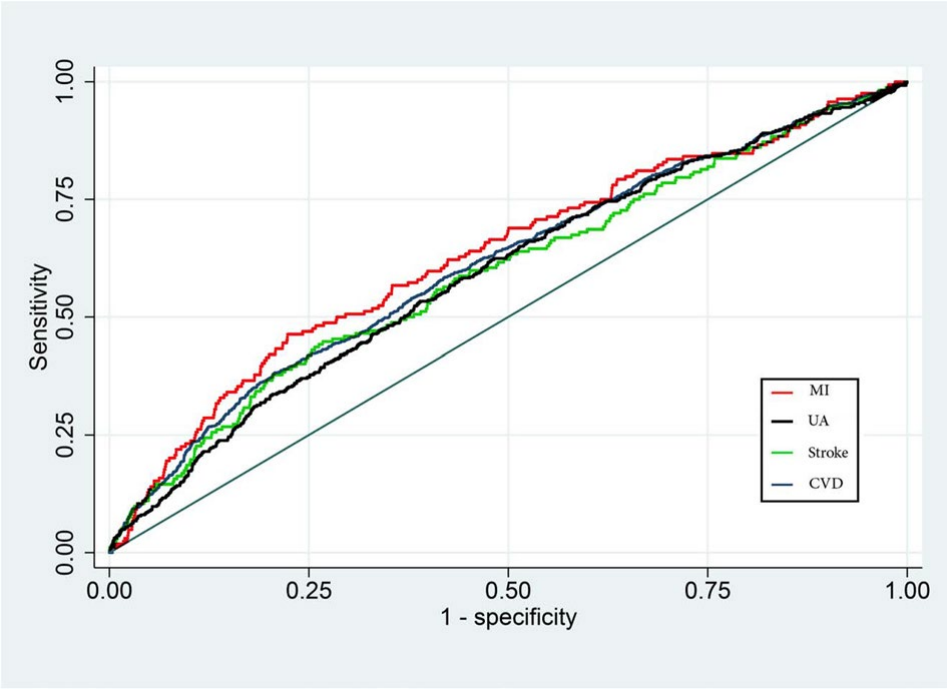
ROC curve analysis of TyG index to predict myocardial infarction (MI), unstable angina (UA), stroke, and cardiovascular disease (CVD)

Table 3 and Fig. 1 present the diagnostic accuracy of the TyG index in predicting CVD outcomes, as evaluated by ROC curve analysis. The AUC in predicting MI was the highest among others as it was 0.631 (95% CI 0.585–0.677). The optimal TyG index cutoff for MI was determined to be 9.04, which provided a sensitivity of 57% and a specificity of 65%. For UA, the AUC was 0.594 (95% CI 0.564–0.623) with an optimal TyG index cutoff of 8.98, yielding a sensitivity of 53% and a specificity of 61%. Regarding overall cardiovascular disease, the TyG index showed an AUC of 0.611 (95% CI 0.590–0.633), with an optimal cut-off point of 8.91, offering a balanced sensitivity and specificity of 58%. When evaluating stroke outcomes, the AUC was 0.595 (95% CI 0.549–0.641), with a sensitivity and specificity observed at 57% for the cut-off point of 8.92.

**Table 3:** The capacity of TyG index to predict CVD outcomes

	AUC^a^	Optimal cut point^b^	Sensitivity (%)	Specificity (%)
Cardiovascular disease	0.611 (0.590–0.633)	8.91	58	58
Myocardial infarction	0.631 (0.585–0.677)	9.04	57	65
Stroke	0.595 (0.549–0.641)	8.92	57	57
Unstable angina	0.594 (0.564–0.623)	8.98	53	61

^a^Area under curve

^b^Derived from Liu method

## Discussion

Within this prospective study, which had a median follow-up period of 11 years, we examined the relationship between the TyG index and the risk of CVD events. Our findings revealed that patients with higher TyG levels exhibited a significantly greater risk for CVD and MI, even after adjusting for potential confounders. These results suggest that the TyG index could serve as a predictive marker for CVD occurrence.

The TyG index has been proposed as a dependable and straightforward surrogate marker for IR. Previous studies have consistently demonstrated its association with IR [6], and robust evidence supports its accuracy [8, 10]. In patients with IR, the physiological response involving the uptake of plasma glucose by cells, inhibition of lipolysis, and endogenous glucose production is disrupted despite the normal levels of plasma insulin [32, 33]. Consequently, de novo lipogenesis contributes to elevated plasma lipid levels during the early stages of IR, even before the onset of hyperglycemia [34]. Therefore, the TyG index, derived from FPG and TG levels, has been proposed as an alternative marker of IR.

The observed increased risk of CVD events associated with higher TyG index levels is consistent with findings from previous studies [12, 15, 35–37]. A 10-year cohort study involving 5014 participants reported a 2.32-fold higher risk of developing CVD in individuals with the highest quintile of the TyG index than in those with the lowest quintile [15]. Another cohort study with 6078 participants showed that the third and fourth quartiles of the TyG index were associated with 1.33 and 1.72 times higher risks of CVD events, respectively [12]. Similarly, a retrospective cohort study conducted using the Korean National Health Information Database showed an increased risk of CVD events in patients in the fourth quartile of the TyG index compared to those in the first quartile [38]. Consistent findings were also observed in previous Iranian studies, where an increased TyG index was associated with higher risks of acute coronary syndrome and cardiac death (1.362-fold and 2.3-fold increase, respectively) in a 6-year prospective cohort study of 9704 healthy participants [18]. Additionally, in another study with a longer follow-up duration in 7521 Iranians, the TyG index showed a strong association with an increased risk of CVD/coronary heart disease, particularly among younger individuals [17]. However, our study is superior to the earlier ones in some ways. For illustration, in comparison with both studies, we recruited participants from both rural and urban areas of three districts in central Iran, while the earlier studies were restricted to a specific metropolitan city. Therefore, the external validity of our findings is greater than that of previous findings. In addition, in comparison with the Mashhad cohort study, we followed participants for a longer duration and identified more events that empowered the associations found in our study. Finally, we adjusted for the confounding effect of diet quality, which is a known determinant of serum lipids and glucose and can potentially affect the associations, whereas two previous studies failed to control for it.

TyG index showed a sufficient discriminative capacity to predict the development of MI and CVD. But, it bordered between bad and sufficient for stroke and UA, as the AUCs were very close to 0.6. This implies that this index may be useful for initial screenings or combined with other factors for predictions but should not be relied on in isolation for a definitive diagnosis. The identified cut-off value for CVD is comparable with the one suggested in a previous study in an Iranian population [17], which suggested a sensitivity of 59.23% and a specificity of 63.15% for the cut-off value of 9.03.

While the discriminative power of the TyG index is moderate to low in our study for the prediction of various cardiovascular outcomes, this is in accordance with findings from other populations and for different endpoints. For example, in a study with patients suffering from diabetes and acute coronary syndrome, an optimal TyG index cut-off of 9.323 had a similarly modest ability to predict major adverse cardiovascular events with an AUC of 0.560, which is close to our AUC for cardiovascular disease (0.611) and myocardial infarction (0.631). The sensitivity in this study was lower (46.0%) compared to our findings (57–58%) but with a comparable specificity (63.6%) [39]. Furthermore, when the TyG index was used to predict the risk of hypertension in a cohort study, the observed sensitivity and specificity (57.85% and 55.40%, respectively) were again comparable to our values for stroke (57% for both) and unstable angina (53% sensitivity and 61% specificity) [40]. Notably, a study in a Turkish population that examined the TyG index’s predictive potential for long-term cardiovascular events showed a higher AUC of 0.71, with better sensitivity (65%) and specificity (63%), suggesting variability in the index’s predictive power based on the population and endpoint assessed [41]. Lastly, a cross-sectional study found a cut-off of 8.44 for subclinical coronary artery disease which delivered a sensitivity of 47.9% and specificity of 68.5%, showing an AUC similar to that for stroke in our study (0.600). These comparisons highlight a consistent pattern where the TyG index appears to offer some predictive utility, but not robustly, reinforcing our suggestion of its use in conjunction with other indices or clinical factors rather than as a standalone diagnostic tool.

Although numerous studies have established a link between an elevated TyG index and a heightened risk of CVD events, the underlying mechanisms behind this relationship are not completely understood. IR has been reported to be associated with both CVD risk factors and incident CVD [42, 43]. It can cause abnormal glucose metabolism, chronic hyperglycemia, and disrupted lipid metabolism. These conditions contribute to chronic inflammation, oxidative stress, and endothelial dysfunction, ultimately leading to cellular damage and atherosclerosis [42, 44, 45]. Based on these facts, it is plausible to propose that IR may serve as an underlying mechanism linking the TyG index to CVD events.

## Strengths and limitations

The current study exhibits several notable strengths. It employs a prospective cohort design coupled with a lengthy follow-up period, allowing for a robust examination of associations over time. Additionally, the study leverages a substantial sample size, encompassing diverse rural and urban regions across three distinct districts in Iran. Consequently, the findings possess enhanced generalizability to other population groups within the country. Nonetheless, certain limitations should be acknowledged. First, the study lacks measurements of fasting plasma insulin, precluding an assessment of the TyG index's concordance with gold standard methods for IR evaluation, such as HIEC and HOMA-IR. However, prior evidence supports the TyG index as a reliable surrogate for measuring insulin resistance, particularly in large-scale population-based studies where direct measurement of IR becomes impractical [10, 46]. Second, the study does not account for changes in the TyG index over time and its potential association with the outcomes of interest. It is plausible that a transition from higher to lower TyG index levels could mitigate the risk of CVD development. Nonetheless, the significant associations observed in this study suggest that even in the event of such a shift, the risk of CVD development remains substantial. Finally, despite adjustments for some relevant confounding variables, the observational nature of the study leaves room for the presence of residual confounders.

## Conclusion

Our study uncovered a robust association between the TyG index and an elevated risk of CVD events over a follow-up period exceeding ten years. These findings underscore the clinical relevance of monitoring the TyG index and its potential as a predictive tool for identifying individuals at risk of CVD development.

## Data Availability

The datasets generated and/or analyzed during the current study are not publicly available due to the policies of Isfahan Cardiovascular Research Centre but are available from the corresponding author on reasonable request.

## Abbreviations

TyG: Triglyceride glucose
CVD: Cardiovascular disease
ICS: Isfahan Cohort Study
MI: Myocardial infarction
UA: Unstable angina
ROC: Receiver operating characteristics
AUC: Area under the curve
IR: Insulin resistance
HIEC: Hyperinsulinemic-euglycemic clamp
HOMA-IR: Homeostasis model assessment of IR
FPG: Fasting plasma glucose
IPAQ: International Physical Activity Questionnaire
FFQ: Food frequency questionnaire
HDL-C: High-density lipoprotein cholesterol
LDL-C: Low-density lipoprotein cholesterol
BMI: Body mass index
GDI: Global dietary index
SBP: Systolic blood pressure
DBP: Diastolic blood pressure
2hPG: 2-Hour postprandial glucose
CK: Creatine kinase
CK-MB: Creatine kinase-myoglobin binding
CK-MBm: CK-MB mass
cTn: Troponin
ANOVA: Analysis of variance
HRs: Hazard ratios
CIs: Confidence intervals
